# Personalized medicine in old age psychiatry and Alzheimer’s disease

**DOI:** 10.3389/fpsyt.2024.1297798

**Published:** 2024-05-01

**Authors:** Nikias Siafarikas

**Affiliations:** Department of Geriatric Psychiatry, Akershus University Hospital, Lørenskog, Norway

**Keywords:** Alzheimer’s disease, personalized medicine, dementia, old age psychiatry, precision medicine

## Abstract

Elderly patients show us unfolded lives with unique individual characteristics. An increasing life span is associated with increasing physical and mental disease burden. Alzheimer’s disease (AD) is an increasing challenge in old age. AD cannot be cured but it can be treated. The complexity of old age and AD offer targets for personalized medicine (PM). Targets for stratification of patients, detection of patients at risk for AD or for future targeted therapy are plentiful and can be found in several omic-levels.

PM aims to «give the right treatment to the right person at the right time» (1). «Personalized medicine», «precision medicine» and «stratified medicine» are lately being used synonymously (2, 3). PM assumes considerable variations between patient groups based on clinical, genetic and environmental characteristics (4). These characteristics are thought to be targets for treatment and prevention and can be found in various domains, including clinical, biological but also social or environmental domains. These domains are usually called “omics” (e.g. genomics, epigenomics, proteomics, metabolomics, microbiomics and so on) (5). Omics give us information we can use to widen the clinical phenotype (**Figure 1**). While in PM patients are stratified and then treated based on various characteristics, the traditional approach in medicine usually applies a “one size fits all treatment” (5, 6).

**Figure 1:**
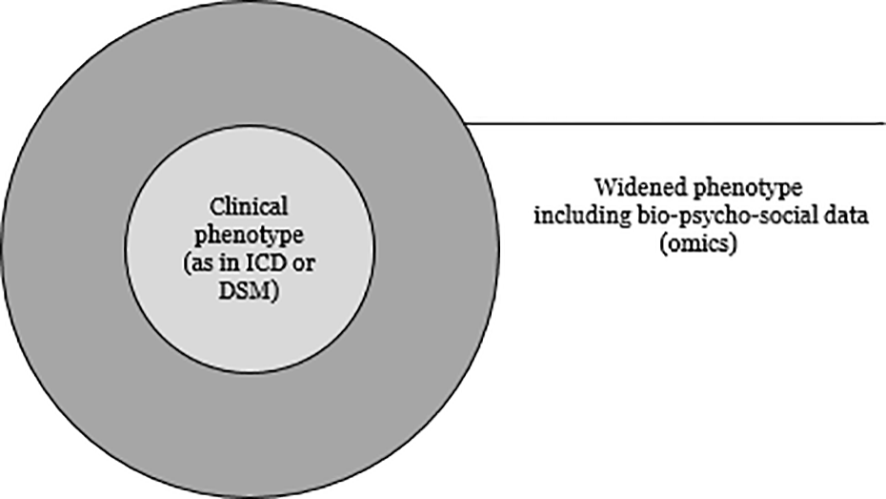
The clinical phenotype needs to be widened with omics data to identify latent subgroups and PM targets.

Old age psychiatry with Alzheimer’s disease (AD) as a prominent challenge appears as an ideal environment for PM [see also (7, 8)]. Major challenges in old age psychiatry include both the differential diagnostic separation from dementia especially against depression and somatic comorbidities, which can contribute to considerable variations between patients. The clinical phenotype alone is often misleading because virtually all psychiatric symptoms are transnosological: cognitive symptoms appear in major depression (9), affective and other non-cognitive symptoms appear in AD with high frequencies (10). Patients in old age psychiatry present “unfolded lives” with the opportunity to identify unique biographical and environmental characteristics.

However, pubmed search results on «personalized medicine» show a steep increase of studies into personalized medicine in the last approximately 10 years with over 100.000 findings (**Figure 2**), but a systematic literature research into “personalized medicine and old age psychiatry” in the same time span showed roughly 300 publications. This apparently stresses the importance of more research in the field. Recently, the Norwegian government published a strategy paper on personalized medicine in Norway from 2023 to 2030 (11), but fails to address dementia and Alzheimer’s disease.

**Figure 2:**
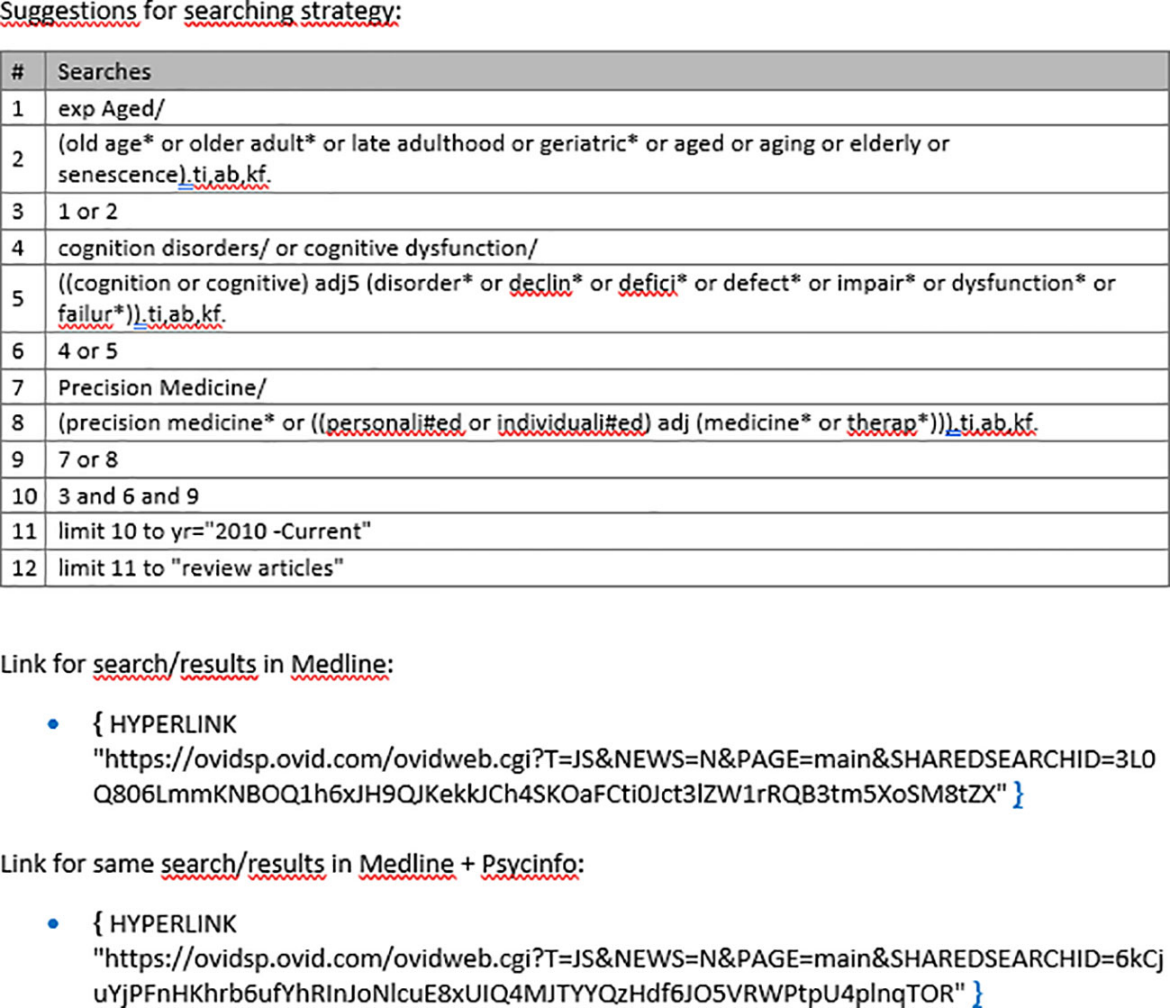
Systematic literature search.

Alzheimer’s disease accounts for approximately 80% of dementia cases (12). AD cannot be cured as of now, but PM may help to better treat it. Biological hallmarks associated to AD appear in various omics levels and include amyloid plaques around the cells, tangles in the cells, loss of synaptic function, neuroinflammation and cholinergic dysfunction (13–15). However, these biological hallmarks of AD are rather indirectly connected to various omics levels and probably represent a phenotype of the highly complex underlying disorder (16). Ultimately, a vicious circle is established including amyloid plaques, intracellular tangles, loss of synaptic function, neuroinflammation and cholinergic dysfunction.

Mounting evidence suggests that the enteral microbiome («microbiomics») is associated with AD («guts to brain axis») (17). Modifiable and non-modifiable risk factors for AD have been found. Modifiable risk factors include nutrition, physical inactivity, smoking, alcohol, obesity, hypertension, diabetes, depression, hearing loss or air pollution; non-modifiable risk factors are basically genetic risk factors such as Apolipoprotein E4 (ApoE4) (18) and age. ApoE4 is associated to several omics levels, including lipid metabolism, vascular function, Amyloid β clearance, glucose metabolism, neurogenesis and synaptic function (19). Metabolites of these mechanisms can be used as biomarkers (e.g. Ab42) (20), but their role as targets for PM is still unclear. AD typical biomarker changes can be detected already in a predementia phase (21), and the definition of preclinical AD is debated (22, 23). Ideally, we would begin AD treatment in this preclinical phase, targeted at specific biological characteristics or e.g. modifiable risk factors. The effect of aging as a risk factor for AD may increase with age (24). The “hallmarks of aging” (25), including genomic instability, epigenetic effects, alterations in protein metabolism and cellular communication, are highly interacting with each other and on the one hand contribute to increasing structural and functional cellular damage and disease burden (26–28), but have on the other hand also been reported as possible targets for PM (29).

Some studies into PM in AD and old-age psychiatry showed promising results. Hearing loss can be a significant risk factor for late-life depression (30), which in turn can be a risk factor for AD (31). Treating comorbid hearing loss can improve depression, thus hearing-loss can be a target in the treatment of LLD and subsequently reduce the risk for AD. Interestingly, when stratified for physical comorbidity, a subgroup of LLD patients with more physical comorbidity benefitted most when hearing loss was treated (32). Another study found considerable biological differences in 500 elderly patients with a diagnosis “major depression” (33). They found two distinct groups with common anatomical and genetic characteristics. Another study examined cerebrospinal fluid and found AD typical changes in approximately 50% of a sample of patients with late life depression (34). Such findings encourage to ask if the “one size fits all treatment” is optimal and provide possible targets for intervention and stratification. Pharmacogenetic testing utilizes differences in genetic profiles and may help identify and stratify patients based on genetic characteristics prior to therapy with psychotropic substances. This is already routine in some places (cyp testing) and is expected to give information if a patient’s genetic profile will yield the optimal response to a drug and to avoid unnecessary “trial and error” treatment. However, evidence for robust clinical usefulness is still slim and genetically guided trials have been suggested (35). Conflicting results have been found regarding Ab plaques as targets for autoantibodies in trials with monoclonal autoantibodies (MAB). Some authors conclude that continuous failures in studies raise doubts if amyloid beta plaques are the right target after all (36), however, they refer to later AD stages and existing Ab plaques. Another study showed a significant slowdown of cognitive decline under treatment with adecanumab which again targets amyloid plaques (37). On the other hand, the newer aducanumab trials, ENGAGE and EMERGE (38), showed side effects of aducanumab in the form of Amyloid-related imaging abnormalities (ARIA), which are mostly asymptomatic but can include headache, seizures or death (39). ARIA have robustly been associated to APOE4 carrier status with APOE4 carriers more likely to experience ARIA under treatment with aducanumab (40) but also other MAB (41–43). Genotyping for APOE4 status is recommended before initializing aducanumab treatment (44) to improve risk assessment, which again is an example for PM patient stratification based on genetic information. In a different approach, the formation of Ab plaques is targeted, the authors (45) conclude that some models with these substances seem promising but admit that «bench to bedside» is not yet reality. Other studies explore substances that aim at tau protein. Some try antibodies in animal models, others try to avoid those structural changes that make tau protein toxic. Although in theory promising, the authors conclude that is uncertain if drugs, targeting tau, help clinically, since there are too few and conflicting studies (46).

Alzheimers disease is associated with neuroinflammation (47). Several studies have reported that exercise reduces neuroinflammation by increasing the expression of anti-inflammatory cytokines. A plausible step – the authors suggest - could be to target neuroinflammation in individualized therapeutic strategies (48). A possible way may be the implementation of strength exercise in personalized therapy regimens to delay AD dementia onset (49). Myokine for instance is secreted by skeletal muscles during exercise, has an anti-inflammatory effect, supports neurogenesis and vascularization and it decreases Amyloid beta production and cell death (50).

Other authors suggest personalized nutrition. Studies have shown increased populations of pro-inflammatory enterobacteria (51). Active metabolites from these bacteria lead to epigenetic changes and may support the formation of amyloid Plaques in the brain. A possible treatment strategy would be personalized nutrition with specific components that can modify the gut microbiome and epigenetic changes. Patients would need to be stratified according to their gut microbiome and their epigenetic signature (52). Interestingly, southern Italians with ApoE4 that lived in Italy – with Mediterranean diet and environment - were in the oldest 1% of the healthy population, but southern Italians with ApoE4 who moved to the United States – different diet, different environment - showed a highly reduced chance of living into this oldest one percent of the healthy population (53). In an example for a successful pilot project (54), patients with Alzheimer’s disease dementia or mild cognitive impairment were treated for nine months with a personalized protocol including cognitive assessment (every 3 months); the PM targets were on various omic levels and included “blood glucose, lipids, inflammation, pathogens, nutrition and autoimmunity; the team included a health coach, a nutritionist, a physical trainer and a physician; nutrition was plant-rich, high-fiber, with salad, fish sometimes eggs and meat, no processed food, simple carbohydrates including gluten and dairy; aerobic and strength training were conducted several times a week; sleep hygiene was supported; stress management provided”. The results indicated cognitive improvement in all groups. A major limitation, though, is the small sample-size (25 included patients). In a multidomain lifestyle intervention approach to reduce cognitive impairment the “Finnish Geriatric Intervention Study to Prevent Cognitive Impairment and Disability (FINGER)” (55–57) targeted several modifiable risk factors by applying “five fingers” daily including a healthy diet, physical, cognitive and social activities as well as the monitoring of cardiovascular risk factors. Paradoxically, the included PM interventions (“five fingers”) did not modify the intervention response, but showed an overall beneficial effect on cognition irrespective of the baseline patient characteristics. Thus, although a beneficial effect of “five fingers” daily has been reported, we still lack information as to which patient or patient group responds best to which intervention.

But old age psychiatry is more than AD. Depression in late life is a major concern with poor treatment outcome (58). Anxiety is another major concern and it has been stressed the importance to include psychotherapy in treatment (59), something that is often challenging given the organizational circumstances in outpatient clinics. Loneliness is crucial and should be addressed and targeted in elderly, “precise interventions that focus on individuals’ needs and the subjective burden of loneliness in the ageing context” have been suggested (60).

Some authors (61) provide an example of how a preclinical, personalized medicine approach in old age psychiatry could look like. They suggest a physical examination with blood tests for Alzheimer biomarkers each year, starting in middle age, including a genetic test for AD risk at the age of 50. If results are positive, further tests for other associated mechanisms would follow including cognitive testing to assess memory impairment. Patients with pathological biomarkers, cognitive impairment or both would be treated early with the most appropriate drug, get neuroimaging examination for precise staging and then receive a drug targeted for their individual etiology and stage of AD. Taken together, a personalized medicine approach in old age psychiatry with an overarching aim to prevent dementia, would target several modifiable risk factors in early stages or preferably in preclinical stages of AD and stratify patients based on biological characteristics including genetic risk factors and epigenetics. Although we cannot cure AD for now, we might be able to delay the onset of dementia and thereby prolong the health span. It has been speculated that a 5-years delay in AD dementia onset would reduce its prevalence with nearly 50% (6). The socioeconomic impact would be tremendous, considering 120 million estimated AD dementia patients in 2050 (62). Some studies are promising in theory, but the clinical significance may still be debatable. Challenges in PM include the transformation of individual clinical assessment into large amounts of data that would in turn form the basis for individual and personalized treatment (3). Evidence based methods including standardized tests would need to be implemented in clinical routines and to be digitalized. A framework has been proposed including evidence based methods and testing for the implementation of PM: the Alzheimer Precision Medicine Initiative (APMI) (63, 64) is built on “4 pillars” (prediction, prevention, personalization and participation, “P4”) including the use of multimodal biomarkers to guide routines, digital devices and health technologies to collect and record patient data as well as data science to analyze and integrate data. Electronic health records (EHR) and portable digital devices such as smart phones or watches equipped with apps can facilitate automated data flow including the registration of longitudinal data (65–68). Data science is needed to create large data bases (3, 64). These data bases would comprise information from multiple omics levels, where artificial intelligence (AI) would be needed to integrate these highly complex data to establish clinically meaningful models (69–73). However, it is crucial to define whose and which data would be relevant to be collected, e. g. from healthy or diseased persons (4, 74). And of course, the protection of data privacy would need to be guaranteed.

## Author contributions

NS: Writing – original draft, Writing – review & editing.

## References

[1] Abrahams E Ginsburg GS Silver M. The Personalized Medicine Coalition: goals and strategies. Am J Pharmacogenomics. (2005) 5:345–55. doi: 10.2165/00129785-200505060-00002 16336000

[2] Wium-Andersen IK Vinberg M Kessing LV McIntyre RS. Personalized medicine in psychiatry. Nord J Psychiatry. (2017) 71:12–9. doi: 10.1080/08039488.2016.1216163 27564242

[3] Goetz LH Schork NJ. Personalized medicine: motivation, challenges, and progress. Fertil Steril. (2018) 109:952–63. doi: 10.1016/j.fertnstert.2018.05.006 PMC6366451 29935653

[4] König IR Fuchs O Hansen G von Mutius E Kopp MV. What is precision medicine? Eur Respir J. (2017) 50. doi: 10.1183/13993003.00391-2017 29051268

[5] Hampel H Nisticò R Seyfried NT Levey AI Modeste E Lemercier P. Omics sciences for systems biology in Alzheimer’s disease: state-of-the-art of the evidence. Ageing Res Rev. (2021) 69:101346. doi: 10.1016/j.arr.2021.101346 33915266

[6] Behl T Kaur I Sehgal A Singh S Albarrati A Albratty M. The road to precision medicine: Eliminating the “One Size Fits All” approach in Alzheimer’s disease. BioMed Pharmacother. (2022) 153:113337. doi: 10.1016/j.biopha.2022.113337 35780617

[7] Cholerton B Larson EB Quinn JF Zabetian CP Mata IF Keene CD. Precision medicine: clarity for the complexity of dementia. Am J Pathol. (2016) 186:500–6. doi: 10.1016/j.ajpath.2015.12.001 PMC4816691 26724389

[8] Hampel H Vergallo A Perry G Lista S. The alzheimer precision medicine initiative. J Alzheimers Dis. (2019) 68:1–24. doi: 10.3233/JAD-181121 30814352

[9] World Health Organization. The ICD-10 classification of mental and behavioural disorders diagnostic criteria for research; ICD-10. Geneva: WHO (1993).

[10] Lyketsos CG Carrillo MC Ryan JM Khachaturian AS Trzepacz P Amatniek J. Neuropsychiatric symptoms in Alzheimer’s disease. Alzheimers Dement. (2011) 7:532–9. doi: 10.1016/j.jalz.2011.05.2410 PMC3299979 21889116

[11] Helsedirektoratet. Nasjonal strategi for persontilpasset medisin 2023–2030. Helsedirektoratet: Oslo. (2023).

[12] Garre-Olmo J. [Epidemiology of Alzheimer’s disease and other dementias]. Rev Neurol. (2018) 66:377–86. doi: 10.33588/rn.6611.2017519 29790571

[13] Scheltens P Blennow K Breteler MM de Strooper B Frisoni GB Salloway S. Alzheimer’s disease. Lancet. (2016) 388:505–17. doi: 10.1016/S0140-6736(15)01124-1 26921134

[14] Ballard C Gauthier S Corbett A Brayne C Aarsland D Jones E. Alzheimer’s disease. Lancet. (2011) 377:1019–31. doi: 10.1016/S0140-6736(10)61349-9 21371747

[15] Lane CA Hardy J Schott JM. Alzheimer’s disease. Eur J Neurol. (2018) 25:59–70. doi: 10.1111/ene.13439 28872215

[16] Cacabelos R Cacabelos P Torrellas C. Handbook of pharmacogenomics and stratified medicine. Elsevier/Academic Press (2014).

[17] Drljača J Milošević N Milanović M Abenavoli L Milić N. When the microbiome helps the brain-current evidence. CNS Neurosci Ther. (2023) 29(Suppl 1):43–58. doi: 10.1111/cns.14076 36601680 PMC10314113

[18] Livingston G Sommerlad A Orgeta V Costafreda SG Huntley J Ames D. Dementia prevention, intervention, and care. Lancet. (2017) 390:2673–734. doi: 10.1016/S0140-6736(17)31363-6 28735855

[19] Safieh M Korczyn AD Michaelson DM. ApoE4: an emerging therapeutic target for Alzheimer’s disease. BMC Med. (2019) 17:64. doi: 10.1186/s12916-019-1299-4 30890171 PMC6425600

[20] Blennow K Zetterberg H. Biomarkers for Alzheimer’s disease: current status and prospects for the future. J Intern Med. (2018) 284:643–63. doi: 10.1111/joim.12816 30051512

[21] Jack CR Jr. Alzheimer disease: new concepts on its neurobiology and the clinical role imaging will play. Radiology. (2012) 263:344–61. doi: 10.1148/radiol.12110433 PMC3329271 22517954

[22] Jack CR Jr. Bennett DA Blennow K Carrillo MC Dunn B Haeberlein SB. NIA-AA Research Framework: Toward a biological definition of Alzheimer’s disease. Alzheimers Dement. (2018) 14:535–62. doi: 10.1016/j.jalz.2018.02.018 PMC5958625 29653606

[23] McKhann GM Knopman DS Chertkow H Hyman BT Jack CR Jr Kawas CH. The diagnosis of dementia due to Alzheimer’s disease: recommendations from the National Institute on Aging-Alzheimer’s Association workgroups on diagnostic guidelines for Alzheimer’s disease. Alzheimers Dement. (2011) 7:263–9. doi: 10.1016/j.jalz.2011.03.005 PMC3312024 21514250

[24] GBD 2019 Diseases and Injuries Collaborators. Global burden of 369 diseases and injuries in 204 countries and territories, 1990-2019: a systematic analysis for the Global Burden of Disease Study 2019. Lancet. (2020) 396:1204–22. doi: 10.1016/s0140-6736(20)30925-9 PMC7567026 33069326

[25] López-Otín C Blasco MA Partridge L Serrano M Kroemer G. Hallmarks of aging: An expanding universe. Cell. (2023) 186:243–78. doi: 10.1016/j.cell.2022.11.001 36599349

[26] Polidori MC. Aging hallmarks, biomarkers, and clocks for personalized medicine: (re)positioning the limelight. Free Radic Biol Med. (2024) 215:48–55. doi: 10.1016/j.freeradbiomed.2024.02.012 38395089

[27] Partridge L Deelen J Slagboom PE. Facing up to the global challenges of ageing. Nature. (2018) 561:45–56. doi: 10.1038/s41586-018-0457-8 30185958

[28] Estimation of the global prevalence of dementia in 2019 and forecasted prevalence in 2050: an analysis for the Global Burden of Disease Study 2019. Lancet Public Health. (2022) 7:e105–25. 10.1016/S2468-2667(21)00249-8 PMC8810394 34998485

[29] Skou ST Mair FS Fortin M Guthrie B Nunes BP Miranda JJ. Multimorbidity. Nat Rev Dis Primers. (2022) 8:48. doi: 10.1038/s41572-022-00376-4 35835758 PMC7613517

[30] Rutherford BR Brewster K Golub JS Kim AH Roose SP. Sensation and psychiatry: linking age-related hearing loss to late-life depression and cognitive decline. Am J Psychiatry. (2018) 175:215–24. doi: 10.1176/appi.ajp.2017.17040423 PMC5849471 29202654

[31] Bennett S Thomas AJ. Depression and dementia: cause, consequence or coincidence? Maturitas. (2014) 79:184–90. doi: 10.1016/j.maturitas.2014.05.009 24931304

[32] Brewster KK Zilcha-Mano S Wallace ML Kim AH Brown PJ Roose SP. A precision medicine tool to understand who responds best to hearing aids in late-life depression. Int J Geriatr Psychiatry. (2022) 37. doi: 10.1002/gps.5721 PMC9942910 35499363

[33] Wen J Fu CHY Tosun D Veturi Y Yang Z Abdulkadir A. Characterizing heterogeneity in neuroimaging, cognition, clinical symptoms, and genetics among patients with late-life depression. JAMA Psychiatry. (2022) 79(5):464–74. doi: 10.1001/jamapsychiatry.2022.0020 PMC8908227 35262657

[34] Siafarikas N Kirsebom BE Srivastava DP Eriksson CM Auning E Hessen E. Cerebrospinal fluid markers for synaptic function and Alzheimer type changes in late life depression. Sci Rep. (2021) 11:20375. doi: 10.1038/s41598-021-99794-9 34645914 PMC8514484

[35] Luzum JA Petry N Taylor AK Van Driest SL Dunnenberger HM Cavallari LH. Moving pharmacogenetics into practice: it’s all about the evidence! Clin Pharmacol Ther. (2021) 110:649–61. doi: 10.1002/cpt.2327 PMC8376790 34101169

[36] Zampar S Wirths O. Immunotherapy Targeting Amyloid-β Peptides in Alzheimer’s Disease. In: Huang X, editor. Alzheimer’s Disease: Drug Discovery. Exon Publications Copyright: The Authors, Brisbane (AU (2020). 33400461

[37] Sevigny J Chiao P Bussière T Weinreb PH Williams L Maier M. The antibody aducanumab reduces Aβ plaques in Alzheimer’s disease. Nature. (2016) 537:50–6. doi: 10.1038/nature19323 27582220

[38] Budd Haeberlein S Aisen PS Barkhof F Chalkias S Chen T Cohen S. Two randomized phase 3 studies of aducanumab in early alzheimer’s disease. J Prev Alzheimers Dis. (2022) 9:197–210. doi: 10.14283/jpad.2022.30 35542991

[39] Cummings J. Anti-amyloid monoclonal antibodies are transformative treatments that redefine alzheimer’s disease therapeutics. Drugs. (2023) 83:569–76. doi: 10.1007/s40265-023-01858-9 PMC10195708 37060386

[40] Loomis SJ Miller R Castrillo-Viguera C Umans K Cheng W O'Gorman J. Genome-wide association studies of ARIA from the aducanumab phase 3 ENGAGE and EMERGE studies. Neurology. (2024) 102:e207919. doi: 10.1212/WNL.0000000000207919 38165296 PMC11097767

[41] Ostrowitzki S Lasser RA Dorflinger E Scheltens P Barkhof F Nikolcheva T. A phase III randomized trial of gantenerumab in prodromal Alzheimer’s disease. Alzheimers Res Ther. (2017) 9:95. doi: 10.1186/s13195-017-0318-y 29221491 PMC5723032

[42] Sperling R Salloway S Brooks DJ Tampieri D Barakos J Fox NC. Amyloid-related imaging abnormalities in patients with Alzheimer’s disease treated with bapineuzumab: a retrospective analysis. Lancet Neurol. (2012) 11:241–9. doi: 10.1016/S1474-4422(12)70015-7 PMC4063417 22305802

[43] Carlson C Siemers E Hake A Case M Hayduk R Suhy J. Amyloid-related imaging abnormalities from trials of solanezumab for Alzheimer’s disease. Alzheimers Dement (Amst). (2016) 2:75–85. doi: 10.1016/j.dadm.2016.02.004 27239538 PMC4879647

[44] Cummings J Rabinovici GD Atri A Aisen P Apostolova LG Hendrix S. Aducanumab: appropriate use recommendations update. J Prev Alzheimers Dis. (2022) 9:221–30. doi: 10.14283/jpad.2022.34 PMC9169517 35542993

[45] Jokar S Khazaei S Gameshgoli XE Khafaji M Yarani B Sharifzadeh M. Amyloid β-Targeted Inhibitory Peptides for Alzheimer’s Disease: Current State and Future Perspectives. In: Huang X, editor. Alzheimer’s Disease: Drug Discovery. Exon Publications Copyright: The Authors, Brisbane (AU (2020). 33400460

[46] Pluta R Ułamek-Kozioł M. Tau Protein-Targeted Therapies in Alzheimer’s Disease: Current State and Future Perspectives. In: Huang X, editor. Alzheimer’s Disease: Drug Discovery. Exon Publications Copyright: The Authors, Brisbane (AU (2020). 33400463

[47] Scheltens P De Strooper B Kivipelto M Holstege H Chételat G Teunissen CE. Alzheimer’s disease. Lancet. (2021) 397:1577–90. doi: 10.1016/S0140-6736(20)32205-4 PMC8354300 33667416

[48] Hampel H Caraci F Cuello AC Caruso G Nisticò R Corbo M. A path toward precision medicine for neuroinflammatory mechanisms in alzheimer’s disease. Front Immunol. (2020) 11:456. doi: 10.3389/fimmu.2020.00456 32296418 PMC7137904

[49] Valenzuela PL Castillo-García A Morales JS de la Villa P Hampel H Emanuele E. Exercise benefits on Alzheimer’s disease: State-of-the-science. Ageing Res Rev. (2020) 62:101108. doi: 10.1016/j.arr.2020.101108 32561386

[50] Lee B Shin M Park Y Won SY Cho KS. Physical exercise-induced myokines in neurodegenerative diseases. Int J Mol Sci. (2021) 22:1–34. doi: 10.3390/ijms22115795 PMC8198301 34071457

[51] Megur A Baltriukienė D Bukelskienė V Burokas A. The microbiota-gut-brain axis and alzheimer’s disease: neuroinflammation is to blame? Nutrients. (2020) 13(1):1–24. doi: 10.3390/nu13010037 PMC7824474 33374235

[52] Milošević M Arsić A Cvetković Z Vučić V. Memorable food: fighting age-related neurodegeneration by precision nutrition. Front Nutr. (2021) 8:688086. doi: 10.3389/fnut.2021.688086 34422879 PMC8374314

[53] Gurinovich A Andersen SL Puca A Atzmon G Barzilai N Sebastiani P. Varying effects of APOE alleles on extreme longevity in european ethnicities. J Gerontol A Biol Sci Med Sci. (2019) 74:S45–s51. doi: 10.1093/gerona/glz179 31724059 PMC7330482

[54] Toups K Hathaway A Gordon D Chung H Raji C Boyd A. Precision medicine approach to alzheimer’s disease: successful pilot project. J Alzheimers Dis. (2022) 88:1411–21. doi: 10.3233/JAD-215707 PMC9484109 35811518

[55] Rosenberg A Ngandu T Rusanen M Antikainen R Bäckman L Havulinna S. Multidomain lifestyle intervention benefits a large elderly population at risk for cognitive decline and dementia regardless of baseline characteristics: The FINGER trial. Alzheimers Dement. (2018) 14:263–70. doi: 10.1016/j.jalz.2017.09.006 29055814

[56] Rosenberg A Mangialasche F Ngandu T Solomon A Kivipelto M. Multidomain interventions to prevent cognitive impairment, alzheimer’s disease, and dementia: from FINGER to world-wide FINGERS. J Prev Alzheimers Dis. (2020) 7:29–36. doi: 10.14283/jpad.2019.41 32010923 PMC7222931

[57] Kivipelto M Mangialasche F Snyder HM Allegri R Andrieu S Arai H. World-Wide FINGERS Network: A global approach to risk reduction and prevention of dementia. Alzheimers Dement. (2020) 16:1078–94. doi: 10.1002/alz.12123 PMC9527644 32627328

[58] Alexopoulos GS. Mechanisms and treatment of late-life depression. Transl Psychiatry. (2019) 9:188. doi: 10.1038/s41398-019-0514-6 31383842 PMC6683149

[59] Penninx BW Pine DS Holmes EA Reif A. Anxiety disorders. Lancet. (2021) 397:914–27. doi: 10.1016/S0140-6736(21)00359-7 PMC9248771 33581801

[60] Akhter-Khan SC Au R. Why loneliness interventions are unsuccessful: A call for precision health. Adv Geriatr Med Res. (2020) 2(3):1–24. doi: 10.20900/agmr20200016 PMC9410567 36037052

[61] Di Meco A Vassar R. Early detection and personalized medicine: Future strategies against Alzheimer’s disease. Prog Mol Biol Transl Sci. (2021) 177:157–73. doi: 10.1016/bs.pmbts.2020.10.002 PMC8641915 33453940

[62] Wimo A Guerchet M Ali GC Wu YT Prina AM Winblad B. The worldwide costs of dementia 2015 and comparisons with 2010. Alzheimers Dement. (2017) 13:1–7. doi: 10.1016/j.jalz.2016.07.150 27583652 PMC5232417

[63] Hampel H Gao P Cummings J Toschi N Thompson PM Hu Y. A Precision Medicine Initiative for Alzheimer’s disease: the road ahead to biomarker-guided integrative disease modeling. Climacteric. (2017) 20:107–18. doi: 10.1080/13697137.2017.1287866 28286989

[64] Hampel H O'Bryant SE Durrleman S Younesi E Rojkova K Escott-Price V. The foundation and architecture of precision medicine in neurology and psychiatry. Trends Neurosci. (2023) 46:176–98. doi: 10.1016/j.tins.2022.12.004 PMC10720395 36642626

[65] Tortelli R Rodrigues FB Wild EJ. The use of wearable/portable digital sensors in Huntington’s disease: A systematic review. Parkinsonism Relat Disord. (2021) 83:93–104. doi: 10.1016/j.parkreldis.2021.01.006 33493786 PMC7957324

[66] Espay AJ Hausdorff JM Sánchez-Ferro Á Klucken J Merola A Bonato P. A roadmap for implementation of patient-centered digital outcome measures in Parkinson’s disease obtained using mobile health technologies. Mov Disord. (2019) 34:657–63. doi: 10.1002/mds.27671 PMC6520192 30901495

[67] Mc Carthy M Schueler P. Editorial: can digital technology advance the development of treatments for alzheimer’s disease? J Prev Alzheimers Dis. (2019) 6(4):217–20. doi: 10.14283/jpad.2019.32 31686090

[68] Hampel H Au R Mattke S van der Flier WM Aisen P Apostolova L. Designing the next-generation clinical care pathway for Alzheimer’s disease. Nat Aging. (2022) 2:692–703. doi: 10.1038/s43587-022-00269-x 37118137 PMC10148953

[69] Wilkinson J Arnold KF Murray EJ van Smeden M Carr K Sippy R. Time to reality check the promises of machine learning-powered precision medicine. Lancet Digit Health. (2020) 2:e677–80. doi: 10.1016/S2589-7500(20)30200-4 PMC9060421 33328030

[70] Myszczynska MA Ojamies PN Lacoste AMB Neil D Saffari A Mead R. Applications of machine learning to diagnosis and treatment of neurodegenerative diseases. Nat Rev Neurol. (2020) 16:440–56. doi: 10.1038/s41582-020-0377-8 32669685

[71] Jordan MI Mitchell TM. Machine learning: Trends, perspectives, and prospects. Science. (2015) 349:255–60. doi: 10.1126/science.aaa8415 26185243

[72] Dipietro L Gonzalez-Mego P Ramos-Estebanez C Zukowski LH Mikkilineni R Rushmore RJ. The evolution of Big Data in neuroscience and neurology. J Big Data. (2023) 10:116. doi: 10.1186/s40537-023-00751-2 37441339 PMC10333390

[73] Wang T Lei Y Fu Y Wynne JF Curran WJ Liu T. A review on medical imaging synthesis using deep learning and its clinical applications. J Appl Clin Med Phys. (2021) 22:11–36. doi: 10.1002/acm2.13121 PMC7856512 33305538

[74] Ramaswami R Bayer R Galea S. Precision medicine from a public health perspective. Annu Rev Public Health. (2018) 39:153–68. doi: 10.1146/annurev-publhealth-040617-014158 29166244

